# Non-destructive Phenotyping of Lettuce Plants in Early Stages of Development with Optical Sensors

**DOI:** 10.3389/fpls.2016.01985

**Published:** 2016-12-27

**Authors:** Ivan Simko, Ryan J. Hayes, Robert T. Furbank

**Affiliations:** ^1^U.S. Department of Agriculture, Agricultural Research Service, Crop Improvement and Protection Research Unit, SalinasCA, USA; ^2^High Resolution Plant Phenomics Centre, Australian Plant Phenomics Facility, Commonwealth Scientific and Industrial Research Organisation Agriculture and Food, CanberraACT, Australia; ^3^Australian Research Council Centre of Excellence for Translational Photosynthesis, Plant Science Division, Research School of Biology, Australian National University, ActonACT, Australia

**Keywords:** temperature stress, elevated salinity, relative chlorophyll content, relative anthocyanin content, photosynthesis, relative growth rate, visual rating of color intensity

## Abstract

Rapid development of plants is important for the production of ‘baby-leaf’ lettuce that is harvested when plants reach the four- to eight-leaf stage of growth. However, environmental factors, such as high or low temperature, or elevated concentrations of salt, inhibit lettuce growth. Therefore, non-destructive evaluations of plants can provide valuable information to breeders and growers. The objective of the present study was to test the feasibility of using non-destructive phenotyping with optical sensors for the evaluations of lettuce plants in early stages of development. We performed the series of experiments to determine if hyperspectral imaging and chlorophyll fluorescence imaging can determine phenotypic changes manifested on lettuce plants subjected to the extreme temperature and salinity stress treatments. Our results indicate that top view optical sensors alone can accurately determine plant size to approximately 7 g fresh weight. Hyperspectral imaging analysis was able to detect changes in the total chlorophyll (RCC) and anthocyanin (RAC) content, while chlorophyll fluorescence imaging revealed photoinhibition and reduction of plant growth caused by the extreme growing temperatures (3 and 39°C) and salinity (100 mM NaCl). Though no significant correlation was found between *F*_v_*/F*_m_ and decrease in plant growth due to stress when comparisons were made across multiple accessions, our results indicate that lettuce plants have a high adaptability to both low (3°C) and high (39°C) temperatures, with no permanent damage to photosynthetic apparatus and fast recovery of plants after moving them to the optimal (21°C) temperature. We have also detected a strong relationship between visual rating of the green- and red-leaf color intensity and RCC and RAC, respectively. Differences in RAC among accessions suggest that the selection for intense red color may be easier to perform at somewhat lower than the optimal temperature. This study serves as a proof of concept that optical sensors can be successfully used as tools for breeders when evaluating young lettuce plants. Moreover, we were able to identify the locus for light green leaf color (*qLG4*), and position this locus on the molecular linkage map of lettuce, which shows that these techniques have sufficient resolution to be used in a genetic context in lettuce.

## Introduction

Lettuce is an economically valuable, leafy vegetable that is harvested when the plant ‘head’ reaches maturity, or in the early stages of development when plant leaves are cut for ‘baby-leaf’ or ‘spring-mix’ bagged salad (Simko et al., 2014). Plants intended for baby-leaf production are grown in the extremely high densities (7.4 million seeds per hectare) until they reach four- to eight-leaf stage and then their leaves are cut. Because baby-leaf lettuces may be cut repeatedly for multiple harvests (Grahn et al., 2015), they need to regrow rapidly, and consistently produce leaves with the shape, color, texture, and taste attractive to consumers. Environmental conditions, such as temperature or soil salinity, however, can severally affect plant growth. Lettuce is rather sensitive to the elevated levels of salt (NaCl/CaCl_2_) in soil or water. When the decline in yield was compared across several vegetable species, lettuce was classified into the ‘most sensitive’ group (Shannon and Grieve, 1999). The increased sensitivity of plants is accentuated during the early stages of development when elevated concentrations of salt inhibit lettuce growth (Shannon et al., 1983; Xu and Mou, 2015). Similarly, the photosynthesis (Seginer et al., 1991) and the growth (Thompson et al., 1998) of lettuce plants are significantly reduced at both suboptimal and supraoptimal temperatures.

Simple optical sensors, such as photographic cameras, have been used for non-destructive plant phenotyping for a long time (Taubenhaus et al., 1929), but the recent technological progress in the development of digital and spectral cameras together with strides in analytical software made these tools more appealing to plant scientists. Phenotyping with optical sensors can accurately be performed on individual plants, plant organs, or a group of plants in laboratory, growth-chamber, greenhouse, or field conditions (Fiorani and Schurr, 2013; Araus and Cairns, 2014). These sensors can be used individually and operated manually or integrated into a high-throughput, fully automated, imaging systems (White et al., 2012). The objective of the present study was to test feasibility of using non-destructive phenotyping with optical sensors for the evaluations of lettuce plants in early stages of development. We performed the series of experiments to determine if hyperspectral imaging and chlorophyll fluorescence imaging can determine phenotypic changes manifested on lettuce plants that were subjected to the extreme temperature and salinity stress treatments. We also analyzed the relationship between data obtained from visual observations of plant color (a consumer’s perspective) and non-destructive phenotyping with optical sensors. Hyperspectral imaging devices used in our study collect data on electromagnetic radiation reflected by a plant for each pixel of the image. These spectral data can be combined to identify specific characteristics, such as internal structure, chemical composition, physiological status, or a damage that may not be evident in the visible spectrum (Simko et al., 2015a, 2017). In difference from the devices that use hyperspectral imaging, instruments based on chlorophyll fluorescence detect only the light that is re-emitted by chlorophyll after the light of the defined wavelength is directed to a plant. Chlorophyll fluorescence analysis thus provides information about the efficiency of photosynthesis (Maxwell and Johnson, 2000) and can detect photosynthetic response of a tissue to environmental stress (Murata et al., 2007). Both the hyperspectral imaging and the chlorophyll fluorescence imaging are routinely used to analyze plant performance (Furbank and Tester, 2011), but to our knowledge they have previously not been used to analyze the early stages of lettuce development described in the present work. Automated, high-throughput phenotyping with optical sensors is well suited for fast evaluations of a large number of plants, such as mapping populations. In this study, we present the application of hyperspectral imaging for rapid, non-destructive determination of chlorophyll content in plants, and use of these data for mapping of the underlying locus.

## Materials and Methods

### Plant Material

The following lettuce accessions were used in one or more experiments: Annapolis (Ann), Balady Banha (BB), Bibb (Bib), Climax (Cli), Corsair (Cor), Eruption (Eru), Flashy Troutback (FT), Grand Rapids (GR), Green Forest (GF), Green Towers (GT), Ice Cube (IC), Iceberg (Ice), La Brillante (LB), Little Gem (LG), Lolla Rossa (LL), Merlot (Mer), Nansen (Nan), Pavane (Pav), Prizehead (Pri), Red Fox (RF), Red Leaf (RL), Redina (Red), RH08-0464 (RH), Salinas (Sal), SM09A (9A), SM09B (9B), SM13-L6 (L6), SM13-R1 (R1), Tom Thump (ToT), Triple Threat (TT), US96UC23 (US), Valmaine (Val), Winter Marvel (WM), and 56 F_8_ recombinant inbred lines (RILs) randomly selected from the Salinas 88 (S88) × La Brillante population (Hayes et al., 2014; Simko et al., 2015b). Five plants per accession per treatment were used in all experiments. These plants were selected from a larger group of plants to minimize differences in plant size at the beginning of the experiment. In experiment 1 (described below), plants of a different size were selected purposely.

### Growing Conditions

Lettuce seeds were sown into square pots (68 mm × 68 mm, 95 mm depth) containing 1:1 mix of soil and sand. Seedlings were grown in a controlled environment growth chamber with 16 h photoperiod, 400 μmol m^-2^ s^-1^ photosynthetic photon-flux density (PPFD), and constant temperature of 21°C (these conditions are called optimal throughout the text, OPT). Temperature stress was applied by decreasing temperature to 3°C (COLD), or increasing it to 39°C (HOT) for the duration described at individual experiments. Watering of plants at all treatments was performed as needed to keep approximately 70% substrate water content (SWC). SWC was determined by weighing the soil before and after drying (Granier et al., 2006). Salt stress (SALT) was imposed by adding NaCl to irrigation water to obtain salinity concentration of 100 mM.

### Tests on Seedlings

In experiments where cotyledons of lettuce seedlings were evaluated, the seeds were either germinated and grown on a wet filter paper in Petri dishes, or in plastic boxes used for holding pipette tips. The boxes contained the same media as was used for growing plants in pots and the seedlings were kept in a grid using plastic that holds pipette tips (Badger et al., 2009). Seedlings were cultivated in OPT conditions until temperature treatments were applied.

### Visual Observations

Visual estimates of plant color intensity were performed on adaxial leaf surfaces. Green color intensity of lettuce leaves was rated light green, green, and dark green, while intensity of red color was rated as no red, light red, and red. These visual estimates of color intensity were compared to relative measurements of anthocyanin and chlorophyll content obtained from hyperspectral imaging.

### Hyperspectral and Chlorophyll Fluorescence Imaging

Hyperspectral imaging was performed with an A-Series VNIR Micro-Hyperspec Sensor (Headwall Photonics, Fitchburg, MA, USA) with the spectral range from 380 to 1,012 nm. The sensor was attached to a metal frame at the distance of 70 cm from scanned samples together with a broad-spectrum halogen lamp (ProLamp, Analytical Spectral Devices, Boulder, CO, USA). Reflectance calibration was performed using Spectralon SRT-MS-100 reflectance standard (Labsphere, North Sutton, NH, USA) that was placed next to the samples at each scan. Images were capture using XCAP v.3.7 software (EPIX, Buffalo Grove, IL, USA) and analyzed with ImageJ 1.49k software (National Institutes of Health, Bethesda, MD, USA).

Measurements of chlorophyll fluorescence were done with PlantScreen (in tray scanning format) or FluorCam 800MF (both from Photon Systems Instruments, Brno, Czech Republic). The protocol parameters were: camera distance 20 cm, TS 20 ms, shutter 1, sensitivity 52%, super 87.2%, *F*_0_ duration 2 s, *F*_0_ period 200 ms, and pulse duration 800 ms. All measurements were performed after 15 min of dark adaptations. Analyses of fluorescence data were carried out using FluorCam 7 (Photon Systems Instruments, Brno, Czech Republic) and ImageJ 1.49k software. Both hyperspectral imaging and chlorophyll fluorescence imaging scans of plants were taken from a top view only.

### Relative Content of Chlorophyll and Anthocyanin

Relative chlorophyll (RCC) and anthocyanin (RAC) content per leaf area were estimated by previously developed indices. These indices are based on measurements of tissue reflectance (*R*) at the specific wavelengths (shown as subscript) obtained from hyperspectral imaging: $RCC=\left(R_{728}-R_{720}\right)/\left(R_{728}+R_{720}-2\times R_{434}\right)$ (Xue and Yang, 2009) and $RAC=\left[R_{800}\times \left(1/R_{550}-1/R_{700}\right)\right]$ (Merzlyak et al., 2003). The indices showed very strong linear relationship (*R*^2^ > 0.9) with a relative content of total chlorophyll (chlorophyll *a* + *b*) and anthocyanin, respectively (Merzlyak et al., 2003; Xue and Yang, 2009) when tested on a diverse set of samples.

### Extent of Photoinhibition

The extent of photoinhibition due to stress caused by abiotic factors was estimated through the analysis of maximum quantum efficiency of photosystem II (PSII) photochemistry. This parameter was calculated as *F*_v_/*F*_m_, where *F*_m_ is the maximum chlorophyll *a* fluorescence yield in the dark-adapted state, and *F*_v_ is the maximum variable fluorescence in the dark-adapted state (calculated as *F*_m_–*F*_o_), and *F*_o_ is the minimum chlorophyll *a* fluorescence yield in the dark-adapted state (Maxwell and Johnson, 2000).

### Total Projected Leaf Area, Relative Growth Rate, and Fresh Weight

Total projected leaf area (*A*_PT_; Munns et al., 2010) in mm^2^ was determined from the images of chlorophyll fluorescence emission at the *F*_m_ level (Barbagallo et al., 2003). To compare *A*_PT_ values to the plant biomass that was produced above-ground, plants were cut at the soil level and their fresh weight (FW) was determined immediately. Relative growth rate (RGR) was calculated from *A*_PT_ as $RGR=\left(\bar{\ln A_{PT2}}-\bar{\ln A_{PT1}}\right)/\left(t2-t1\right)$, where $\bar{\ln A_{PT1}}$ and $\bar{\ln A_{PT2}}$ are the means of natural logarithm transformed *A*_PT_ at the times *t*_1_ and *t*_2_, respectively (Hoffmann and Poorter, 2002). The values of RGR were multiplied by 100 to obtain units in mm^2^ per cm^2^ per day (mm^2^ × cm^-2^ × d^-1^).

### Description of Individual Experiments

#### Experiment 1: Relationship between *A*_PT_ and FW, and between Visual Observations of Leaf Color and RCC and RAC

Accessions: Ann, Bib, Cli, Eru, FT, GR, GF, GT, Ice, LB, LG, LL, Mer, Pav, RF, RL, RH, Sal, TT, and Val.

Growing conditions: 15 days in OPT.

Evaluations: *A*_PT_, FW, visual observation of red and green leaf color, RCC and RAC; in addition, disks (1 cm diameter) were cut from selected leaves at the end of the experiment to make comparisons of RCC, RAC, and *F*_v_/*F*_m_ at adaxial and abaxial surfaces of the leaves.

Note: Plants of highly different sizes were selected for the analysis of relationship between *A*_PT_ and FW.

#### Experiment 2: Change in Growth and Photosynthesis in Suboptimal Temperature

Accessions: Eru, GR, GT, LB, LG, Pav, RL, Sal, and TT.

Growing conditions: 10 days in OPT, then 10 days in either OPT or COLD.

Evaluations: *A*_PT_ and *F*_v_/*F*_m_.

#### Experiment 3: Change in Growth, Photosynthesis, RAC, and RCC in Suboptimal and Supraoptimal Temperatures

Accessions: 110, 112 (two RILs from the S88 × LB population), 9A, 9B, Eru, GR, GT, LB, LG, Pav, R1, RL, and TT. Five of the accessions (Eru, LB, LG, RL, and TT) were selected for hyperspectral analyses to determine changes in RCC and RAC after the temperature treatment.

Growing conditions: 10 days OPT, then 8 days in either OPT, COLD, or HOT, then 6 days in OPT (recovery).

Evaluations: *A*_PT_, *F*_v_/*F*_m_, RCC, and RAC.

#### Experiment 4: Change in Growth and Photosynthesis under Increased Salinity

Accessions: 9A, GT, LB, LG, Pav, RL, and R1.

Growing conditions: 10 days in OPT, then 8 days in either OPT or SALT.

Evaluations: *A*_PT_ and *F*_v_/*F*_m._

#### Experiment 5: Change in Photosynthesis in Suboptimal Temperature – Seedlings in Petri Dish

Accessions: GR, LB, Sal, and TT.

Growing conditions: 4 days in OPT, then 1 day in either OPT or COLD.

Evaluations: *F*_v_/*F*_m_*_._*

#### Experiment 6: Change in Photosynthesis in Suboptimal Temperature – Seedlings in Plastic Box

Accessions: 9A, 9B, Ann, BB, Bib, Cli, Cor, Eru, FT, GF, GR, GT, IC, Ice, L6, LB, LL, Mer, Nan, Pav, Pri, Red, RF, RH, RL, R1, Sal, ToT, TT, US, Val, and WM.

Growing conditions: 4 days in OPT, then 2 days in COLD.

Evaluations: *F*_v_/*F*_m_.

#### Experiment 7: Detection of RCC and Mapping Locus – Seedlings in Plastic Box

Accessions: 56 RILs from the S88 × LB population.

Growing conditions: 4 days in OPT.

Evaluations: RCC.

Note: Visual observations of leaf color were previously performed on the same RILs grown under field conditions (Hayes et al., 2014; Simko et al., 2015b). The evaluations were performed on adult plants at harvest maturity using the same scale (light green, green, and dark green) as in the experiment 1.

### Statistical Analyses

Differences between means of two groups were tested with *t*-test (or paired *t*-test), and among multiple groups with one-way analysis of variance (ANOVA). If ANOVA results were significant, the Tukey-Kramer HSD test was applied to compare all pairs of groups. The *F*-test was used to test if two variances are equal. The Pearson correlation coefficient was utilized to measure a linear correlation between two variables. All statistical analyses were calculated with JMP v. 11.1.1 (SAS Institute, Cary, NC, USA).

### Quantitative Trail Locus (QTL) Mapping

Quantitative Trail Locus for RCC and green leaf color visually observed on RILs were mapped with QGene v. 4.3.9 software (Joehanes and Nelson, 2008) using simple interval mapping. The significance threshold for QTL scores was determined empirically through permutations with 1,000 iterations (Churchill and Doerge, 1994). Molecular linkage map developed for this population was previously described in detail (Hayes et al., 2014; Simko et al., 2015b).

## Results

**FIGURE 1:**
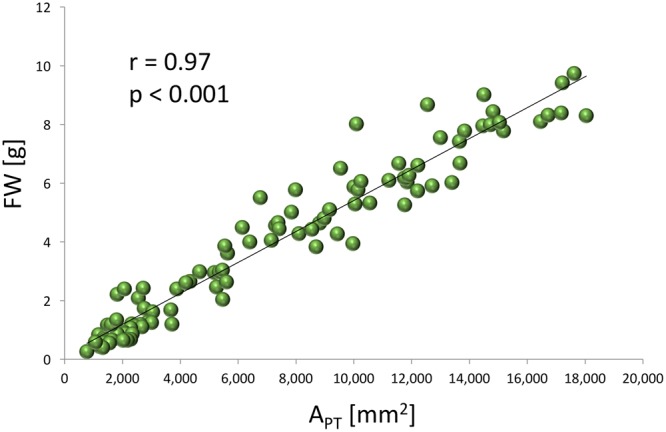
**Relationship between the total projected leaf area (*A*_PT_) determined from the images of chlorophyll fluorescence emission and the fresh weight (FW) of lettuce plants (experiment 1)**.

**FIGURE 2:**
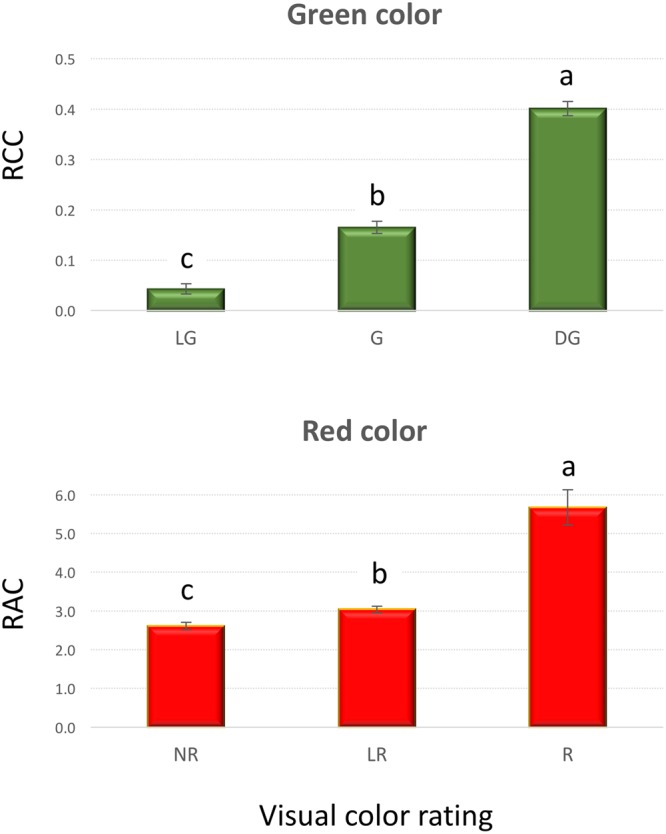
**Relative chlorophyll content (RCC) and relative anthocyanin content (RAC) in plants that were visually rated for the intensity of green (top) and red (bottom) leaf color (experiment 1)**. Vertical bars show standard errors; different letters within each panel indicate means that are significantly different at *p* < 0.05. Visual color rating scale was: LG – light green, G – green, DG – dark green, NR – no red, LR – light red, and R – red.

**FIGURE 3:**
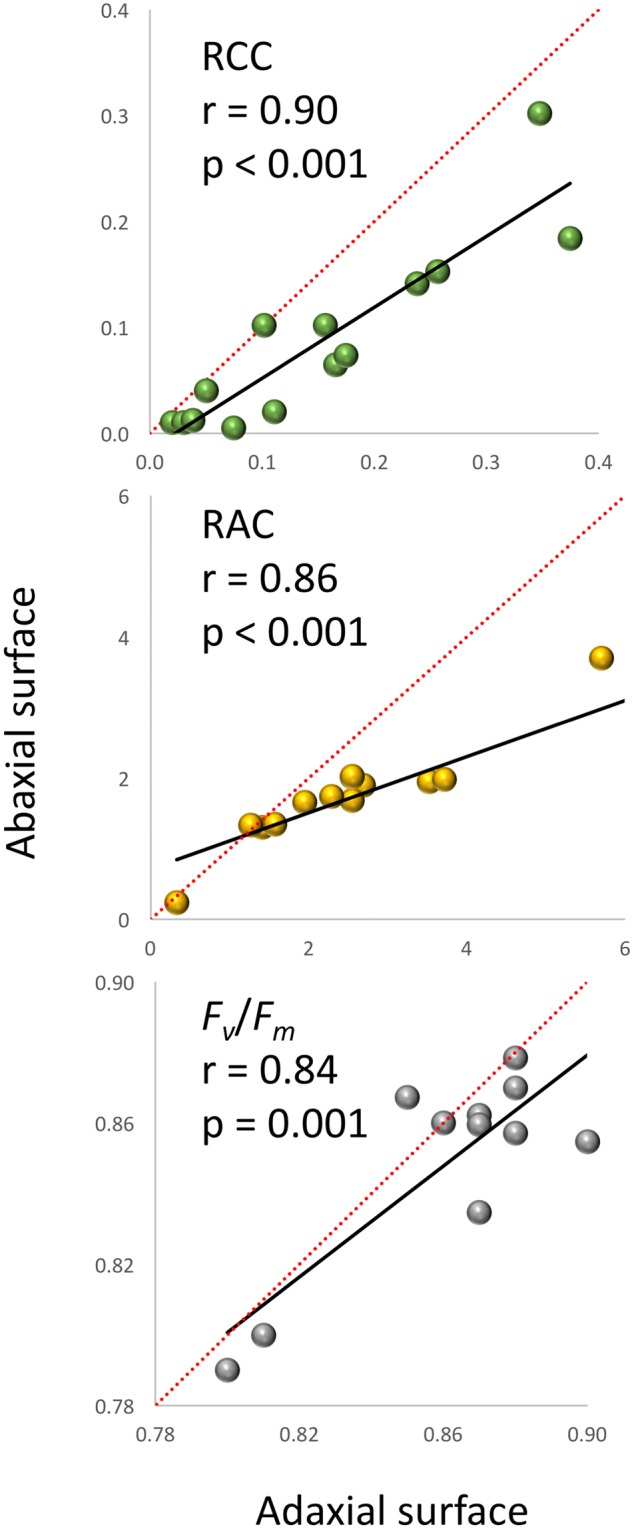
**Relationship between the relative chlorophyll content (RCC), relative anthocyanin content (RAC) and *F*_v_*/F*_m_ measured on the adaxial and abaxial plant leaf surfaces (experiment 1).** The linear regression lines are in black, while the diagonal red lines indicate trends for the parameters if values were the same on both leaf surfaces (1:1 relationship).

A very strong, positive, linear correlation (*r* = 0.97, *p* < 0.0001) was observed between *A*_PT_ and FW (**Figure 1**) in experiment 1, demonstrating that optical sensors can be used to accurately estimate plant above-ground biomass from top view imaging of leaf area in the early stages of lettuce development. Visual classification of leaf color into three green and three red groups was in good agreement with RCC and RAC (**Figure 2**) values calculated from hyperspectral imaging. Differences in RCC among three green groups were highly significant. When RAC values were compared, a relatively small difference was detected between ‘no red’ (mean of 2.61) and ‘light red’ (mean of 3.04) groups, but this difference was also significant at *p* < 0.01. These data indicate that visual classification of green and red leaf color can be used for initial estimates of total chlorophyll and anthocyanin concentration in lettuce leaves, and that the combination of chlorophyll and anthocyanin concentration has a substantial effect on a customer’s visual perception of lettuce leaf color. When the measurements of RCC, RAC, and *F*_v_/*F*_m_ were performed on the adaxial and abaxial surfaces of leaves, a strong, linear correlation was observed between surfaces for each parameter (RCC: *r* = 0.90, *p* < 0.0001; RAC: *r* = 0.86, *p* < 0.0001; *F*_v_/*F*_m_: *r* = 0.84, *p* = 0.001; **Figure 3**). However, there were small, yet consistent differences between values measured on the two surfaces when compared across all tested leaves. The overall values of the parameters were higher on the adaxial surface (RCC: 0.15 vs. 0.09, *p* < 0.001; RAC: 2.72 vs. 1.80, *p* = 0.005; and *F*_v_/*F*_m_: 0.86 vs. 0.85, *p* = 0.037) and the differences between the surfaces were generally more pronounced at greater values of each parameter (**Figure 3**).

**FIGURE 4:**
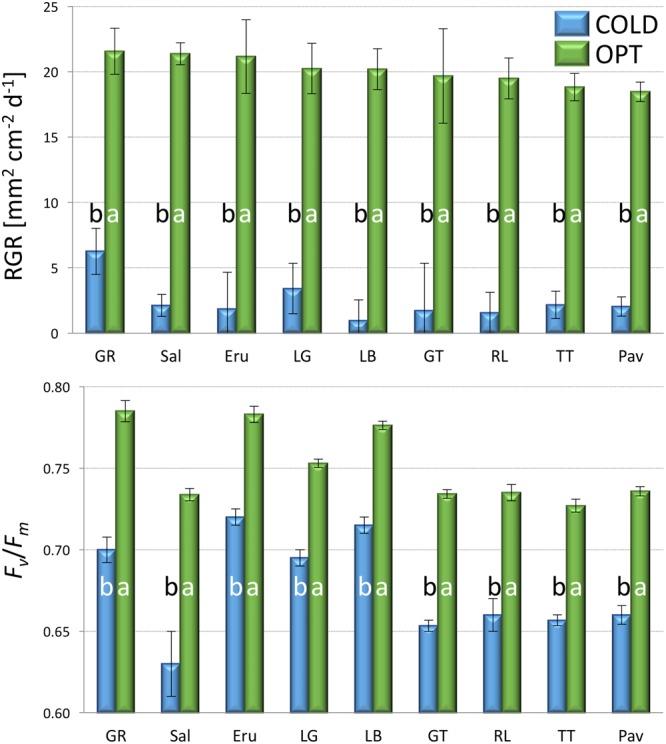
**Relative growth rate (RGR, top) and *F*_v_*/F*_m_ (bottom) of plants cultivated in optimal (OPT, green color) or low (COLD, blue color) temperature (experiment 2).** Vertical bars show standard errors (*n* = 5); different letters within each accession indicate means that are significantly different at *p* < 0.05.

RGR of all accessions significantly decreased when plants were cultivated at COLD conditions (experiment 2). The average RGR for nine tested accessions was 20.1 in OPT, while during the same period of time it was only 2.5 in COLD (**Figure 4**). Similarly, *F*_v_/*F*_m_ significantly decreased for all accession when in COLD, and the overall mean dropped from 0.75 in OPT to 0.68 in COLD (**Figure 4**). No significant correlation was detected between RGR and *F*_v_/*F*_m_ in OPT (*r* = 0.66, *p* = 0.055), or in COLD (*r* = 0.21, *p* = 0.568).

**FIGURE 5:**
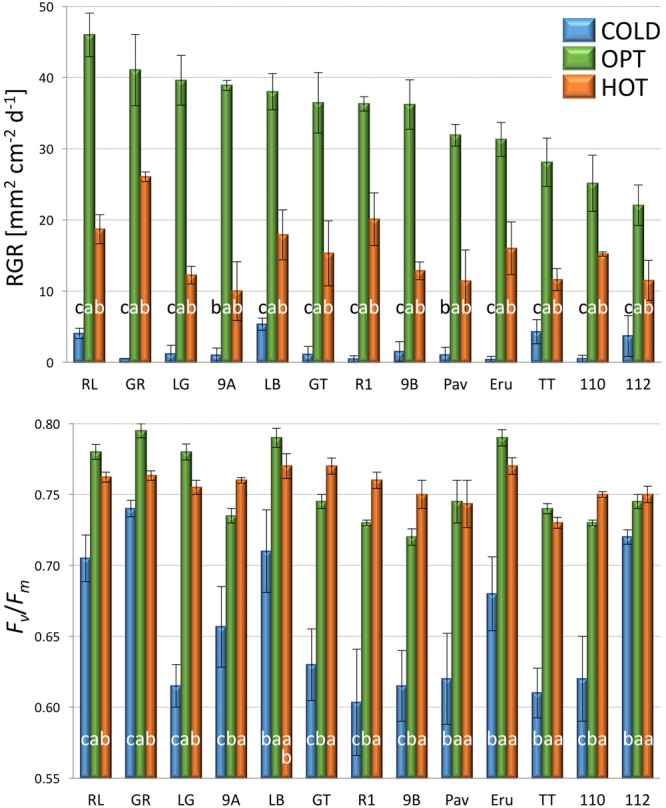
**Relative growth rate (RGR, top) and *F*_v_*/F*_m_ (bottom) of plants cultivated in optimal (OPT, green color), low (COLD, blue color), or high (HOT, orange color) temperature (experiment 3).** Vertical bars show standard errors (*n* = 5); different letters within each accession indicate means that are significantly different at *p* < 0.05.

**FIGURE 6:**
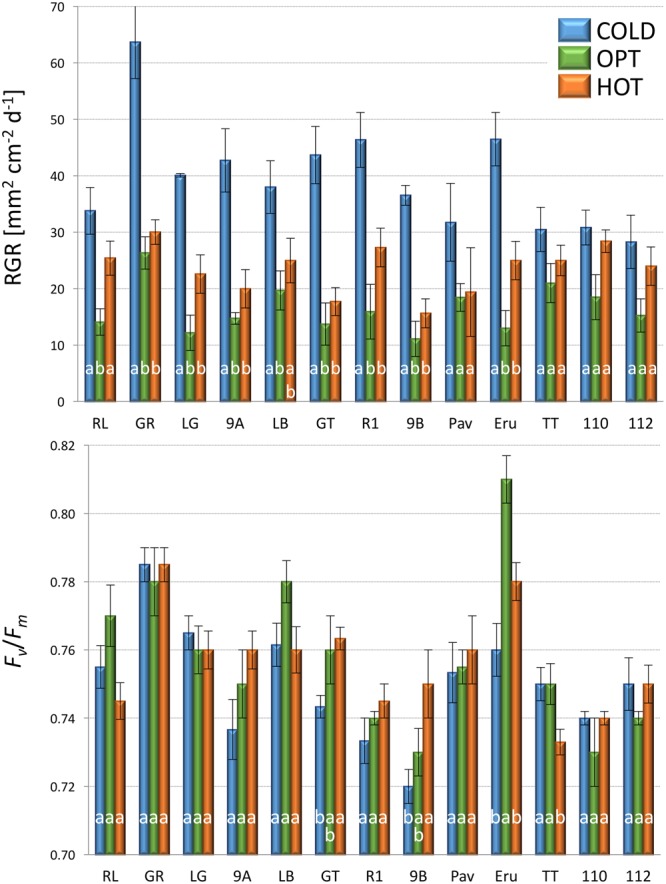
**Relative growth rate (RGR, top) and *F*_v_*/F*_m_ (bottom) of plants constantly cultivated in optimal (OPT, green color) temperature, or transferred to the optimal temperature after 8 days at low (COLD, blue color) or high (HOT, orange color) temperature (experiment 3).** Vertical bars show standard errors (*n* = 5); different letters within each accession indicate means that are significantly different at *p* < 0.05.

**FIGURE 7:**
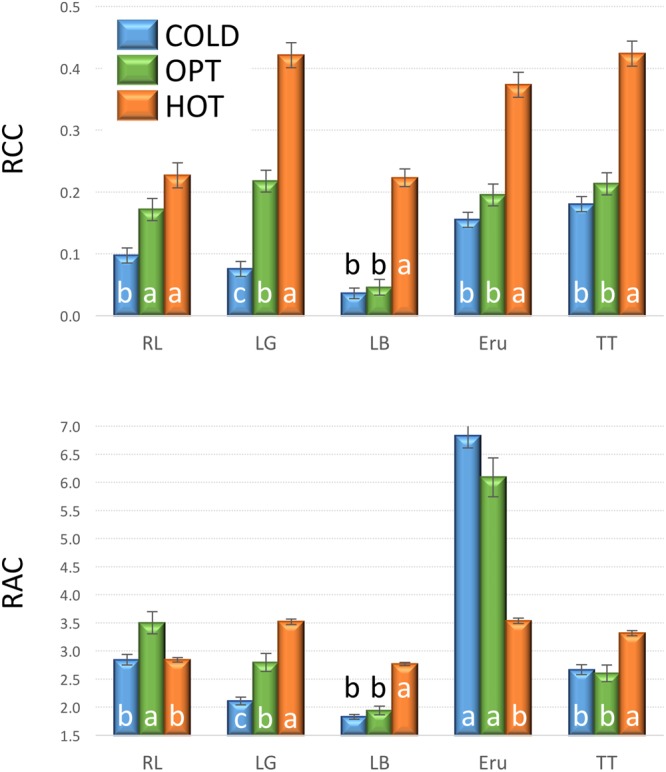
**Relative chlorophyll content (RCC, top) and relative anthocyanin content (RAC, bottom) in plants cultivated in optimal (OPT, green color), low (COLD, blue color), or high (HOT, orange color) temperature (experiment 3).** Vertical bars show standard errors (*n* = 5); different letters within each accession indicate means that are significantly different at *p* < 0.05.

**FIGURE 8:**
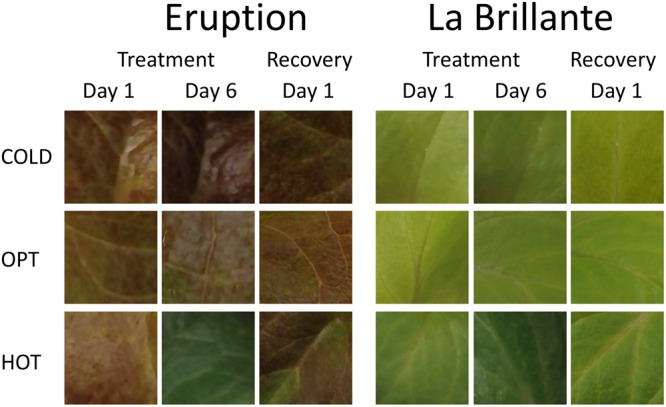
**Changes in the leaf color for the plants cultivated in low (COLD), optimal (OPT), and high (HOT) temperatures.** For each cultivar, the first column shows the leaf color 1 day after transferring plants from OPT to the respective treatments, the second column shows the leaf color after 6 days in different temperatures, and the third column shows the leaf color 1 day after moving plants back to the OPT temperature (recovery period). Notice that after 6 days in HOT both cultivars showed almost the same dark green color, though in OPT and COLD temperatures the two cultivars differ substantially in their leaf color (cv. Eruption is red while cv. La Brillante is light green). The total length of the temperature treatment period was 8 days. The color of temperature-treated plants was getting back to typical already 1 day after moving them to the OPT temperature (column 3). After 6 days of recovery, the color of temperature-treated plants was visually undistinguishable from those constantly kept at OPT temperature (experiment 3).

The growth of plants tested in the experiment 3 was substantially reduced in both low and high temperatures. The overall RGR was 34.7 in OPT, 1.9 in COLD, and 15.3 in HOT. Significant decrease in RGR was detected for all accessions in COLD or HOT when compared to OPT (**Figure 5**). However, while all accessions almost completely stopped growing in COLD, their growth was still noticeable in HOT. Similarly, as in the experiment 2, the *F*_v_/*F*_m_ parameter significantly decreased for all accessions when cultivated in COLD (the overall average at OPT = 0.76 and in COLD = 0.66; **Figure 5**). Though the overall average of *F*_v_/*F*_m_ remained the same in HOT (0.76) as in OPT, the change in this parameter varied widely across accessions. The *F*_v_/*F*_m_ value significantly decreased in HOT compared to OPT in three accessions (GR, LG, and RL), did not change significantly in five accessions (112, Eru, LB, Pav, and TT), and significantly increased in five accessions (110, 9A, 9B, GT, and R1). No significant correlation was detected between *F*_v_/*F*_m_ and RGR in any of the three growing conditions (OPT: *r* = 0.44, *p* = 0.133; COLD: *r* = 0.36, *p* = 0.230; and HOT: *r* = 0.52, *p* = 0.068). After returning plants cultivated in COLD to OPT conditions for 6 days their growth markedly improved (**Figure 6**). The overall RGR of plants constantly grown in OPT was 16.5, while for those previously grown in COLD it was 39.5. Significant increase in RGR (as compared to OPT) was detected for nine out of 13 accessions that were previously in COLD (9A, 9B, Eru, GR, GT, LB, LG, R1, and RL). The change in RGR was not so obvious for plants cultivated in HOT after they were returned to OPT. The overall RGR of these plants was 23.5, and only a single accession (RL) showed significantly higher RGR when compared to the plants constantly cultivated in OPT. The overall *F*_v_/*F*_m_ for plants constantly in OPT was 0.76, for those moved from COLD 0.75, and for those previously in HOT 0.76 (**Figure 6**). These results show that the large drop in *F*_v_/*F*_m_ in COLD (the overall value of 0.66) was not permanent and the plants recovered after moving to OPT. In cv. Eru, however, the *F*_v_/*F*_m_ value for plants constantly in OPT was significantly higher (0.81) than for those moved in from HOT (0.78) or COLD (0.76) conditions. This difference does not seem to be caused by a damage to the light harvesting system but rather by a gradual increase in the *F*_v_/*F*_m_ value for the plants in OPT. It has increased from 0.79 at the end of the temperature treatment period to 0.81 at the end of the recovery period. Again, no significant, linear correlation was detected between RGR and *F*_v_/*F*_m_ after recovery period (OPT: *r* = 0.14, *p* = 0.646; COLD: *r* = 0.45, *p* = 0.123; and HOT: *r* = 0.03, *p* = 0.920). Five selected accession submitted to hyperspectral imaging showed significant changes in RCC and RAC when cultivated under OPT, COLD, and HOT conditions. RCC gradually rose in all accessions with increasing temperature (**Figure 7**). The overall RCC values in COLD were 0.11, in OPT 0.17, and in HOT 0.33. In contrast, the overall RAC levels stayed almost the same in different conditions (COLD = 3.3, OPT = 3.4, and HOT = 3.2). Changes in RAC, however, varied across tested accessions (**Figure 7**). While RAC gradually decreased with the increasing temperature for the accession with the highest level of RAC in COLD (Eru), RAC increased in the accessions that had the lowest RAC in COLD (LG and LB). The changes in RCC and RAC appear to be reversible as readjustments in green and red coloring of foliage were already visually observable 1 day after moving plants from COLD and HOT to OPT conditions (**Figure 8**).

**FIGURE 9:**
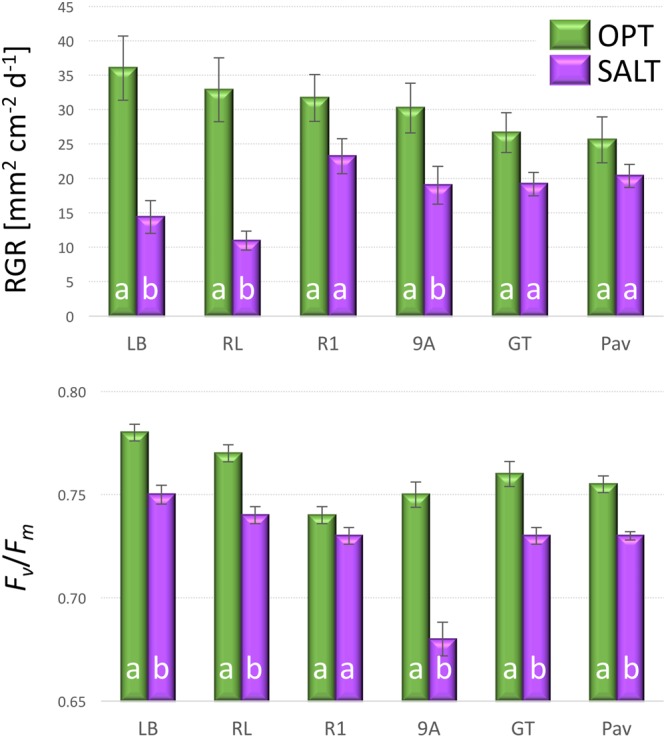
**Relative growth rate (RGR, top) and *F*_v_*/F*_m_ (bottom) of plants cultivated in optimal (OPT, green color) or elevated salinity (SALT, purple color) conditions (experiment 4).** Vertical bars show standard errors (*n* = 5); different letters within each accession indicate means that are significantly different at *p* < 0.05.

The addition of NaCl into irrigation water significantly affected both plant growth (RGR) and the efficiency of photosystem (*F*_v_/*F*_m_). The overall RGR decreased from 30.5 in OPT to 17.9 in SALT (**Figure 9**). Though all accession had lower RGR in SALT, the difference was significant in only three (9A, LB, and RL) out of six tested accessions. Similarly, the *F*_v_/*F*_m_ parameter decreased in SALT for all accessions, though the difference was not significant for R1 (**Figure 9**). The overall value of *F*_v_/*F*_m_ dropped from 0.76 in OPT to 0.73 in SALT. No significant correlation was found between RGR and *F*_v_/*F*_m_ in OPT (*r* = 0.49, *p* = 0.320), SALT (*r* = -0.36, *p* = 0.478), or the drop in the two parameters (*r* = 0.09, *p* = 0.867).

**FIGURE 10:**
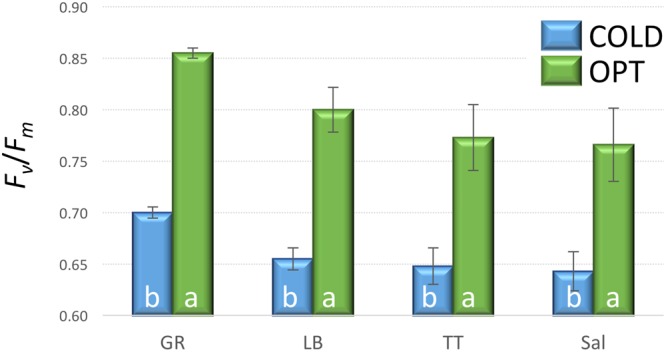
***F*_v_*/F*_m_ on cotyledons of seedlings cultivated in optimal (OPT, green color) or low (COLD, blue color) temperature in Petri dishes (experiment 5).** Vertical bars show standard errors (*n* = 5); different letters within each accession indicate means that are significantly different at *p* < 0.05.

**FIGURE 11:**
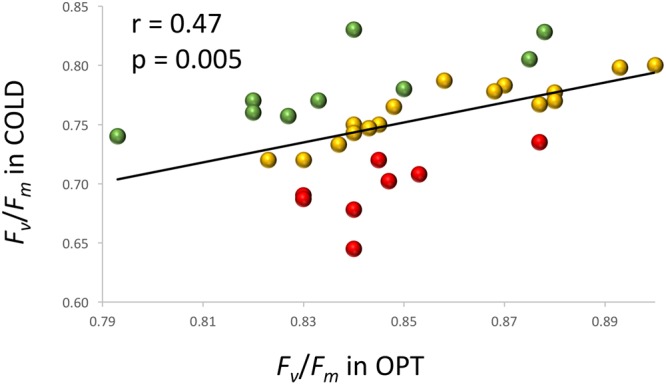
**Relationship between *F*_v_*/F*_m_ measured on cotyledons of seedlings cultivated in optimal (OPT, *x*-axis) or low (COLD, *y*-axis) temperature in plastic boxes (experiment 6).** Red color indicates accessions with the largest decrease (≥0.12) in the *F*_v_*/F*_m_ values, green color indicates accessions with the lowest decrease (≤0.07) in the *F*_v_*/F*_m_ values, and yellow color indicates accessions with the intermediate decrease (0.08–0.11) in the *F*_v_*/F*_m_ values. The *F*_v_*/F*_m_ values in COLD were significantly (*p* ≤ 0.05) smaller for all but one accessions.

When young seedlings cultivated in Petri dishes were transferred to COLD conditions their *F*_v_/*F*_m_ significantly decreased within a day as compared to the seedlings kept at OPT (**Figure 10**). While the overall value of *F*_v_/*F*_m_ for cotyledons in OPT was 0.80, it was only 0.66 in COLD. Similar results were observed on young seedlings of 33 accessions grown in a soil/sand mix in plastic boxes. After 2 days of temperature treatment, the overall *F*_v_/*F*_m_ value in OPT was 0.85, while in COLD it was only 0.75. The *F*_v_/*F*_m_ parameter significantly decreased in all accessions (drop ranged from 0.05 to 0.20) with the exception of cv. ToT, in which a drop of 0.03 occurred that, was not significant at *p* < 0.05. A relatively low decrease of *F*_v_/*F_m_* (≤0.07) was also observed in accessions L6, Cli, Cor, US, BB, WM, Nan, and IC. In contrast, the largest decline in this parameter (≥0.12) was observed in Eru, 9A, R1, RH, RL, Val, Pri, and 9B. There was a weak, but significant correlation (*r* = 0.47, *p* = 0.005) between *F*_v_/*F*_m_ values in OPT and COLD (**Figure 11**).

**FIGURE 12:**
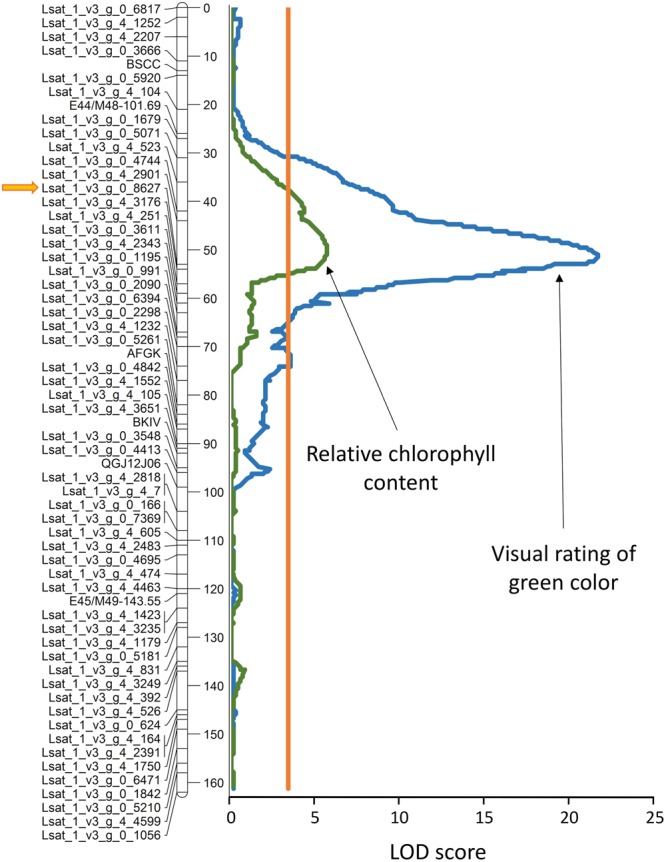
**Genomic position of the quantitative trail locus (QTL) for light green color (*qLG4*) on linkage group 4.** Visual rating of the green color intensity was performed on adult plants in field, while the relative chlorophyll content (RCC) was determined from hyperspectral reflectance measured on cotyledons of seedlings cultivated in plastic boxes (experiment 7). The orange line parallel with the linkage map shows the significance threshold (α = 0.05). The allele for light green color and low RCC originates from cv. La Brillante. Detailed description of the linkage map for this population and its construction was published previously (Hayes et al., 2014; Simko et al., 2015b). Distance in cM is shown on the right site of the linkage map. LOD, logarithm of odds.

Values of RCC measured on cotyledons of 56 randomly selected F_8_ RILs from the S88 × LB population were used to map locations of loci underlying this trait. A single, highly significant QTL (LOD = 5.5) was detected on linkage group 4 (LG 4), tightly linked to the marker Lsat_1_v3_g_0_8627 (**Figure 12**; **Table 1**). Data from the field evaluations of lettuce leaf color on 90 RILs from the same population yielded also only a single QTL (LOD = 21.1) that was located at the same chromosomal region, indicating that the measurements of RCC on cotyledons and the visual assessment of green color on adult plants likely sense the same trait. The QTL for light-green color (*qLG4*) explains 38 and 68% of the total phenotypic variation for RCC and the visual color rating, respectively. The linear correlation between the phenotypic values of the two traits was highly significant (*r* = 0.71, *p* < 0.0001).

**Table 1:** Genomic location of the *qLG4* locus and its effect on the intensity of green color and the relative chlorophyll content.

Trait	No. of RILs^c^	LG	Marker^d^	QTL location (cM)^e^	Support interval (cM)^f^	LOD	R^2^%^g^
Color^a^	90	4	Lsat_1_v3_g_0_8627	51.3	49.3–53.0	21.1	67.6
RCC^b^	56	4	Lsat_1_v3_g_0_8627	49.7	44.5–53.9	5.5	38.4

*^a^Visual evaluation of green color on adult plants in field. ^b^Relative chlorophyll content measured on cotyledons of seedlings grown in plastic boxes. ^c^90 RILs from the S88 × LB population were visually evaluated for the leaf color, but only a subset of 56 RILs was evaluated for RCC. ^d^Molecular marker nearest to the QTL. ^e^Location of the QTL on the molecular linkage map. ^f^The range of 1-LOD support interval. ^g^Percent of the total phenotypic variation of the trait explained by the QTL. The allele for light green color and low RCC originates from cv. La Brillante.*

## Discussion

### Evaluation of Plant Size

Optical sensors that provide a top view image have commonly been used for the non-destructive estimates of leaf area for the plants with planophile growth habit (Barbagallo et al., 2003; Jansen et al., 2009; Flood et al., 2016). We have successfully used chlorophyll fluorescence imaging, rather than color or monochrome images, to estimate *A*_PT_ that in turn accurately predicts FW of young lettuce plants (**Figure 1**). This evaluation is possible because lettuce plants in the early stages of growth have almost planophile growth habit, with relatively flat, non-overlapping leaves. When the plants develop further, their growth habit become more erectophile, with leaves starting to overlap. Therefore, we do not recommend using the top view-based optical sensors alone for the estimates of lettuce size above 7 g FW without multiple view imaging, stereo imaging or other techniques to generate a height dimension.

### Evaluation of Leaf Color

Lettuce leaf color is critically evaluated by customers when making purchasing decisions. The amount and the distribution of leaf pigments contributes to the visual appeal of lettuce; thus it is important to analyze changes in the color under different growing conditions. We have determined that the visual rating of the green and red leaf color is strongly associated with the values of RCC and RAC obtained from hyperspectral imaging (**Figure 2**). Previously, a good correlation (*r* = 0.76) was found between the ratings of lettuce red color intensity performed by human panelists and the direct measurements of anthocyanin levels (Gazula et al., 2007). Panelists, however, could not determine red color in a cultivar with a low anthocyanin level. Similarly, we have detected anthocyanin through hyperspectral analysis in the leaf samples with the ‘no red’ color rating, confirming that the threshold level of anthocyanin needed for the detection by visual observation is higher than is the actual level of anthocyanin in some lettuces (Gazula et al., 2007).

Several previous studies (e.g., Merzlyak et al., 2003; Xue and Yang, 2009) and our present analyses show that hyperspectral imaging can be successfully used for the quantification of chlorophyll and anthocyanin in leaves. There are certain aspects, however, that need to be considered when using optical sensors for such quantifications. The top view optical sensors quantify levels of pigments on the adaxial surface only, while extraction-based methods analyze samples that normally represent the cross section of the leaf. Though we detected very strong, linear correlations between RCC (*r* = 0.90) and RAC (*r* = 0.86) on the abaxial and adaxial leaf surfaces (**Figure 3**), the absolute difference between results obtained for the two surfaces increased as the level of pigments in leaves increased. This expanding difference is caused by a greater increase in pigments on the surfaces that have a direct contact with light. Therefore, scanning of both surfaces with optical sensors may be considered when such analysis is feasible; e.g., at the end of the experiment when plants can be removed from pots or leaves cut. Also, when plants age and their leaves begin overlapping, optical sensors cannot scan the hidden areas of leaves, thus detecting pigments only on the visible areas exposed to light. Nevertheless, the use of optical sensors has several major advantages compared to the quantifications performed on extracts from leaf tissue; it is much faster, it can analyze the whole plant surface (and even multiple plants) at once and promptly identify differences within a leaf, plant or between plants non-destructively, thus allowing analysis of the same leaf over time.

### Effect of Suboptimal Temperature

Our results show that the suboptimal temperatures have a major effect on the RGR, RCC and RAC levels (**Figure 12**), and the plant photochemistry as measured by the *F*_v_/*F*_m_ parameter. When the plants were transferred from OPT to COLD conditions, their growth immediately decreased to almost zero and was followed by a drop in *F*_v_/*F*_m_ and RCC. A similar decline of RGR and *F*_v_/*F*_m_ was previously observed in *Arabidopsis thaliana* L. plants transferred from 22°C/18°C (day/night) to 5°C (Jansen et al., 2009), in *F*_v_/*F*_m_ and chlorophyll content of watermelon plants [*Citrullus lanatus* (Thunb.) Matsum. & Nakai] transferred to 12°C/10°C from 25°C/15°C (Hou et al., 2016), and in *F*_v_/*F*_m_ of lettuce plants submitted to 4°C for 24 h (Oh et al., 2009). When compared to OPT conditions, RAC in COLD decreased in the accessions with low and intermediate RAC levels, but increased in the accession with high RAC (**Figure 7**). After returning plants to the OPT conditions, RGR, RCC, RAC (**Figure 8**), and *F*_v_/*F*_m_ went back to the levels measured prior to the COLD treatment, indicating that the plants were not permanently damaged at 3°C. Remarkably, lettuce plants showed a very high resilience to low temperatures. In a single, unreplicated experiment, plants from each of the accessions tested in the experiment 2 were kept at COLD for 3 months (at 16 h photoperiod and 400 μmol m^-2^ s^-1^ PPFD). After moving plants to OPT they immediately recovered without any obvious damage that could be visually observed (data not shown). Our results indicate that lettuce plants have a high adaptability to temperatures close to freezing, at least under the conditions tested in this study. We did not find any significant relationship between the decrease in RGR and *F*_v_/*F*_m_ at COLD, or after moving plants from COLD to OPT. It was reported previously, that *F*_v_/*F*_m_ decreases faster in the cold-sensitive *A. thaliana* plants than in the less sensitive plants when cultivated at 5°C (Jansen et al., 2009). These authors, however, compared only two genotypes (wild type and transgenic); thus the results may not represent the general trend across many different genotypes. Still, it is possible, that *F*_v_/*F*_m_ values in lettuce may show a relationship to RGR if tested under different environmental conditions (temperature, photoperiod, and/or PPFD).

### Effect of Supra-Optimal Temperature

**FIGURE 13:**
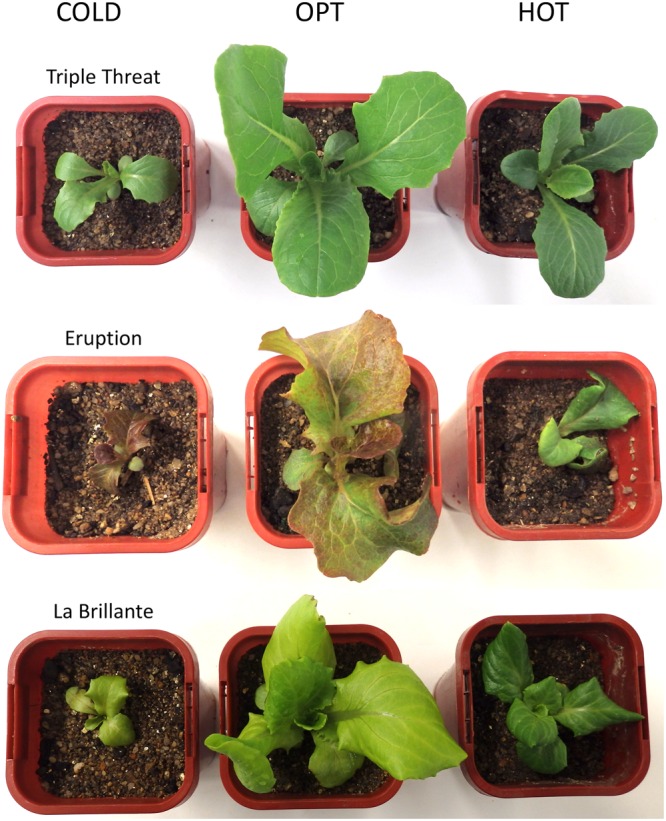
**Comparison of the size and the color of plants cultivated at optimal (OPT), low (COLD), and high (HOT) temperatures (experiment 3).** Plants were initially grown at OPT for 10 days and then either continuously kept in OPT or transferred to COLD or HOT for 8 days. Sides of the square pots are 68 mm long.

The increase in temperature from 21°C (OPT) to 39°C (HOT) led to a significant decrease in RGR and increase in RCC in all accessions (**Figures 5, 7, 8**, and **13**). RAC substantially decreased only in the accession with very high RCC at OPT, but increased in the majority of the accessions with the lower levels of RCC in OPT (**Figures 7, 8**, and **13**). The change in the *F*_v_/*F*_m_ parameter varied greatly among accessions (increasing, staying unchanged, or decreasing; **Figure 5**). A previous study on watermelon reported only a slight drop in *F*_v_/*F*_m_ when temperature increased from 25°C/15°C to 42°C/40°C at the irradiance level of 250 μmol m^-2^ s^-2^ (Hou et al., 2016). When lettuce plants of a single cultivar were exposed to 38°C for 3 h the *F*_v_/*F*_m_ ratio somewhat decreased, but returned to almost the original values 21 h after the treatment (Oh et al., 2009).

Reduced concentrations of chlorophyll and anthocyanin have been observed in lettuce grown at supra-optimal temperatures (Gazula et al., 2005; Chon et al., 2012). These changes in the chlorophyll content are at odds with our observations that show overall increases in RCC with growing temperature (**Figures 7, 8**, and **13**). Similar to our results, almost a 10-fold increase in chlorophyll content has been observed in plants of cultivar Grand Rapids when the average temperature was raised from 23 to 33°C (at 600 μmol m^-2^ s^-1^; Frantz et al., 2004). These large differences between studies may be caused by numerous factors, including accessions used in the studies, other environmental conditions interacting with temperature (photoperiod, PPFD, humidity, etc.), levels of nutrients in the growing substrate, watering regime, and the age of plants. The HOT temperature treatment used in our study (39°C) is probably close to the upper limit that cultivated lettuce could survive when continuously exposed to for several days. In the preliminary test (data not shown) we used the temperature of 42°C that led to severe stress and irreversible modifications in plants, such as chlorotic lesions, malformed leaves, dropping of leaves, and also plant death.

**FIGURE 14:**
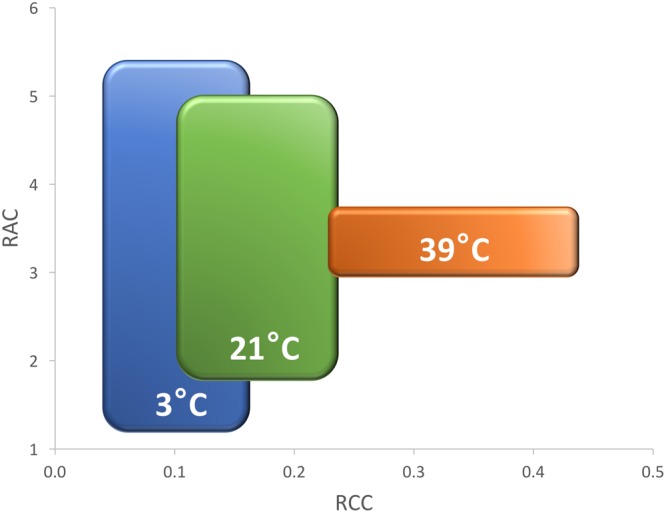
**Effect of increasing temperatures on relative chlorophyll content (RCC, *x*-axis) and relative anthocyanin content (RAC, *y*-axis) in five cultivars (Eruption, Little Gem, La Brillante, Red Leaf, and Triple Threat).** Plants were cultivated in low (3°C, COLD, blue color), optimal (21°C, OPT, green color), and high (39°C, HOT, orange color) temperatures. Rectangles indicate the extend of standard deviations that were calculated from the mean values of all cultivars. The overall mean and the variance of RCC gradually grows with the increasing temperature. Opposite, the overall mean of RAC stays nearly stable while the variance significantly decreases (experiment 3). The large difference in RAC among cultivars in COLD (3°C) suggests that the selection for red color may be performed at somewhat lower than the optimal temperature.

The overall mean of RAC stayed similar across treatments (COLD = 3.26, OPT = 3.39, and HOT = 3.20), while the variance among accessions radically decreased (significantly smallest in HOT; **Figure 14**). Because the RAC variance within accessions did not substantially change under the different temperature treatments, the ANOVA *F*-value was over four times greater for RAC in COLD than in OPT or HOT. Hence, the breeders selecting for dark red color (high RAC) may consider temporarily subjecting lettuce plants to low temperatures where differences among genotypes are likely to be more pronounced (assuming that the pattern of changes in pigments is the same as in our study). These results are somewhat unexpected, because several previous studies reported that low temperatures lead to increased anthocyanin production in lettuce (Gazula et al., 2005, 2007; Boo et al., 2011; Chon et al., 2012; Becker et al., 2014a). However, when the regulation of anthocyanin biosynthesis [quantified as cyanidin-3-*O*-(*6*″-malonyl)-glucoside] was compared in three cultivars, a substantial difference in their response to varying temperatures was detected. While anthocyanin production in the red oak cultivar was negatively correlated with increasing temperature, the correlation was positive in the two Batavia cultivars (Marin et al., 2015). Nevertheless, it is problematic to compare results of temperature treatments from diverse studies, because several factors, including radiation (Tsormpatsidis et al., 2008, 2010; Marin et al., 2015), relative humidity (Marin et al., 2015), water availability (Rajabbeigi et al., 2013), light source (Park et al., 2012), CO_2_ availability (Park et al., 2012), and plant growth stage (Becker et al., 2014b) affect biosynthesis of anthocyanin in lettuce either directly or in interaction. It is possible, that supra-optimal temperature (HOT) in our experiments caused a drought stress, despite regular watering. It was demonstrated that drought stress significantly increases anthocyanin content in lettuce (Rajabbeigi et al., 2013). Therefore, in future experiments, it may be useful to assess also differences in plant transpiration among tested accessions.

In contrast to RAC, the changes in temperature affected RCC in all accessions similarly. The overall mean of RCC grew from 0.11 in COLD, to 0.17 in OPT, to 0.33 in HOT, while the variance also gradually, but non-significantly, increased (**Figure 14**). Differences in RCC among accessions suggest that the selection for higher RCC may be performed at somewhat higher than the optimal temperature, but the change in *F*-value was relatively minor (1.5 higher in HOT than in OPT) compared to that seen in RAC.

### Effect of Elevated Salinity

Elevated salinity inhibits growth of young leaves (the rapid, osmotic phase of the plant response to salt) and accelerates senescence of mature leaves (the slower, ionic phase of the plant response; Munns and Tester, 2008). Beside reducing plant growth, increased concentrations of salt lead to decreased water content in lettuce, lower concentrations of chlorophyll *a* and *b*, smaller intracellular spaces, increased elasticity of leaves, higher concentration of phenolic acids, and larger leaf areas occupied by palisade and spongy parenchyma (Garrido et al., 2014). Our study, focusing on detecting changes in RGR and photochemical efficiency (*F*_v_/*F*_m_), determined that the growth of young plants was substantially reduced after exposing them to 100 mM NaCl for 8 days. The inhibition of plant growth did not significantly correlate with the reduction of *F*_v_/*F*_m_ (**Figure 9**). Slower growth was previously observed for young lettuce plants (Xu and Mou, 2015) that were subjected to a mix of NaCl and CaCl_2_. Similar to our study, the reduction in FW was not correlated with the *F*_v_/*F*_m_ values. These results suggest that *F*_v_/*F*_m_ is not a robust indicator of the performance of lettuce under salt stress, possibly because growth may be more sensitive to the osmotic component of salt stress than photochemical efficiency (Munns and Tester, 2008). The impact of stress on *F*_v_/*F*_m_ is strongly linked to the severity of the stress, as its value has been observed to gradually decrease with increasing concentrations of NaCl applied (Bartha et al., 2010; Qin et al., 2013); potentially due to tissue damage from salt rather than an osmotic effect. Therefore, higher concentrations of NaCl, or a longer exposure to the salinity would likely yield different results.

### Allele for Light Green Leaf Color

At least 10 genes related to the chlorophyll level in lettuce have been described previously (Robinson et al., 1983), including the *lg* gene for light green leaf color. Though the *lg* gene *per se* has not yet been mapped, the gene is loosely linked to the lettuce mosaic virus resistance gene *mo-1* (Ryder, 1992) located on LG 4 (Nicaise et al., 2003; McHale et al., 2009). It is plausible then that *qLG4* (**Figure 12**) may be linked (or is allelic) to the *lg* gene. The *qLG4* locus detected in our study is different from QTLs for the total chlorophyll content that have previously been mapped to LGs 3, 7, and 9 (Damerum et al., 2015), or from those for chlorophyll *a* and chlorophyll *b* content located on LGs 1, 2, and 8 (Hayashi et al., 2012).

## Conclusion

An application of optical sensors for the analysis of plants is getting increased attention from plant scientists and growers, as the cost of sensors decreases and they become to be more widely available. Because sensors are amenable to automatization, high-throughput, automatic phenotyping is particularly attractive for the use in large-scale experiments (White et al., 2012; Fiorani and Schurr, 2013; Araus and Cairns, 2014), performed under field- or environmentally controlled conditions. Sensor-based phenotyping of lettuce is still, however, in only early stages of development. To our knowledge, automatic phenotyping with optical sensors is not yet commonly applied for analysis of lettuce plants in field, though optical sensor-based machines are already commercially used for precise thinning of lettuce crop (Blue River Technology, Sunnyvale, CA, USA). More studies are needed to develop sensing and analytical tools and mathematical models that can be applied for the precise evaluation of lettuce plants in advanced stages of development when leaves from the same or nearby plants overlap and heads (a grouping of tightly packed, overlaying leaves) form. Such phenotyping tools would be valuable for the evaluations of crop development and its overall quality. Small-, and medium-size phenotyping studies performed in environmentally controlled areas (e.g., greenhouse, growth chamber, or laboratory) on young plants can be used by lettuce breeders to evaluate plant growth, architecture, and resistance and to select genotypes with desirable traits at early stages of development.

The present study was designed to test feasibility of using optical sensors for physiological evaluation of lettuce plants in early stages of their development with the long term aim of using these tools for breeding applications. Our results indicate that top view sensors can accurately determine plant size to approximately 7 g FW. Hyperspectral imaging analysis was able to detect changes in the total chlorophyll and anthocyanin levels, while chlorophyll fluorescence imaging revealed photoinhibition and reduction of plant growth caused by the extreme growing temperatures and salinity. Though no significant correlation was found between *F*_v_/*F*_m_ and decrease in plant growth due to stress when comparisons were made across multiple accessions, it is possible that this parameter may be used to determine the level of stress within an accession (e.g., gradual decrease in *F*_v_/*F*_m_ values with falling temperatures) or be useful at higher levels of salt stress. It was demonstrated before that low temperatures, moderate heat, salt stress, and CO_2_ limitation inhibit the repair of PSII by suppressing the synthesis of D1 protein that is required for the assembly of the active PSII complex (Murata et al., 2007). Therefore, more detailed studies are needed to investigate the genotype-specific effect of different stress factors on the decrease of *F*_v_/*F*_m_ in lettuce. This study, however, serves as a proof of concept that optical sensors can be successfully used for non-destructive phenotyping of young lettuce plants. Moreover, we were able to identify the locus for light green leaf color (*qLG4*), and position this locus on the molecular linkage map of lettuce showing that these techniques have sufficient resolution to use in a genetic context in lettuce.

## Author Contributions

IS designed and performed the experiments, statistically analyses and interpreted the data, and wrote the manuscript. RH developed the mapping population, contributed to data interpretation, and revised the manuscript. RF developed approaches for the phenotypic analyses, provided crucial expertise for designing of the experiments and data interpretation, and revised the manuscript.

## Conflict of Interest Statement

The authors declare that the research was conducted in the absence of any commercial or financial relationships that could be construed as a potential conflict of interest.
